# Personality and the use of cancer screenings - Results of the German National Cohort

**DOI:** 10.1016/j.pmedr.2024.102677

**Published:** 2024-03-08

**Authors:** André Hajek, Heiko Becher, Hermann Brenner, Bernd Holleczek, Verena Katzke, Rudolf Kaaks, Heike Minnerup, André Karch, Hansjörg Baurecht, Michael Leitzmann, Annette Peters, Sylvia Gastell, Wolfgang Ahrens, Ulrike Haug, Katharina Nimptsch, Tobias Pischon, Karin B. Michels, Anja Dorrn, Carolina J. Klett-Tammen, Stefanie Castell, Stefan N. Willich, Thomas Keil, Sabine Schipf, Claudia Meinke-Franze, Volker Harth, Nadia Obi, Hans-Helmut König

**Affiliations:** a Department of Health Economics and Health Services Research, University Medical Center Hamburg-Eppendorf, Hamburg Center for Health Economics, Hamburg, Germany; b Heidelberg University Hospital, Heidelberg Institute of Global Health, Heidelberg, Germany; c Division of Clinical Epidemiology and Aging Research, German Cancer Research Center (DKFZ), Heidelberg, Germany; d Saarland Cancer Registry, Saarbrücken, Germany; e Department of Cancer Epidemiology, German Cancer Research Center (DKFZ), Heidelberg, Germany; f Institute for Epidemiology and Social Medicine, Faculty of Medicine, University of Münster, Münster, Germany; g Department of Epidemiology and Preventive Medicine, University of Regensburg, 93053 Regensburg, Germany; h Institute of Epidemiology, Helmholtz Zentrum München - German Research Center for Environmental Health (GmbH), Neuherberg, Germany; i Chair of Epidemiology, Institute for Medical Information Processing, Biometry and Epidemiology, Medical Faculty, Ludwig-Maximilians-Universität München, Munich, Germany; j German Institute of Human Nutrition Potsdam-Rehbruecke, Nuthetal, Germany; k Leibniz Institute for Prevention Research and Epidemiology – BIPS, Bremen, Germany; l Molecular Epidemiology Research Group, Max Delbrück Center for Molecular Medicine in the Helmholtz Association (MDC), Berlin, Germany; m Max Delbrueck Center for Molecular Medicine in the Helmholtz Association (MDC), Biobank Technology Platform, Berlin, Germany; n Charité - Universitaetsmedizin Berlin, Corporate Member of Freie Universitaet Berlin, Humboldt-Universitaet zu Berlin, Berlin, Germany; o Institute for Prevention and Cancer Epidemiology, Faculty of Medicine and Medical Center, University of Freiburg, Freiburg, Germany; p Department for Epidemiology, Helmholtz Centre for Infection Research, Brunswick, Germany; q Institute of Social Medicine, Epidemiology and Health Economics, Charité - Universitätsmedizin Berlin, Berlin, Germany; r Institute of Clinical Epidemiology and Biometry, University of Würzburg, Würzburg, Germany; s State Institute of Health I, Bavarian Health and Food Safety Authority, Erlangen, Germany; t Institute for Community Medicine, Department SHIP/Clinical-Epidemiological Research, University Medicine Greifswald, Greifswald, Germany; u Institute for Occupational and Maritime Medicine, University Medical Center Hamburg-Eppendorf, Hamburg, Germany

**Keywords:** Cancer screening, Personality, Mammography, Prostate cancer, Health screening

## Abstract

**Objective:**

To determine the association between personality characteristics and use of different cancer screenings.

**Methods:**

We used data from the German National Cohort (NAKO; mean age was 53.0 years (SD: 9.2 years)) – a population-based cohort study. A total of 132,298 individuals were included in the analyses. As outcome measures, we used (self-reported): stool examination for blood (haemoccult test, early detection of bowel cancer), colonoscopy (screening for colorectal cancer), skin examination for moles (early detection of skin cancer), breast palpation by a doctor (early detection of breast cancer), x-ray examination of the breast (“mammography”, early detection of breast cancer), cervical smear test, finger examination of the rectum (early detection of prostate cancer), and blood test for prostate cancer (determination of Prostate-Specific Antigen level). The established Big Five Inventory-SOEP was used to quantify personality factors. It was adjusted for several covariates based on the Andersen model. Unadjusted and adjusted multiple logistic regressions were computed.

**Results:**

A higher probability of having a skin examination for moles, for example, was associated with a higher conscientiousness (OR: 1.07, p < 0.001), higher extraversion (OR: 1.03, p < 0.001), higher agreeableness (OR: 1.02, p < 0.001), lower openness to experience (OR: 0.98, p < 0.001) and higher neuroticism (OR: 1.07, p < 0.001) among the total sample. Depending on the outcome used, the associations slightly varied.

**Conclusions:**

Particularly higher levels of extraversion, neuroticism and conscientiousness are associated with the use of different cancer screenings. Such knowledge may help to better understand non-participation in cancer screening examinations from a psychological perspective.

## 1 Introduction

Cancer is among the leading causes of premature death worldwide (Jemal et al., 2008). According to the World Health Organization, around one in three to one in two cancer cases are preventable (World Health Organization, unknown year), e.g., through lifestyle modifications. In addition, the importance of secondary prevention should be stressed – which is the early detection and treatment of such diseases. It is important because the early detection can considerably increase the probability of a successful cure. Common known secondary prevention actions include x-ray examination of the breast or colorectal cancer screening.

Screenings, once proven effective, are typically covered by health insurance in numerous countries, with government agencies actively promoting the utilization of various cancer screenings (e.g., (Bundesministerium für Gesundheit, 2023, Cancer Research UK, 2022, Centers for Disease Control and Prevention, 2023). However, similar to other nations, the participation rate in cancer screenings remains relatively low in Germany (Spuling et al., 2016).

Factors influencing cancer screenings can be categorized according to the Andersen model of healthcare utilization (Andersen, 1995). That theoretical model delineates determinants into predisposing characteristics (such as age group or sex), enabling resources (such as type of health insurance or income) and need factors (such as chronic illnesses or self-rated health). For example, prior research has shown that several predisposing characteristics (e.g., classic factors such as marital status (Bremer et al., 2019) and also more rarely investigated factors such as religion (Kretzler et al., 2020)) and need factors (Gonzalez et al., 2012, Shah et al., 2022) are associated with the probability of using cancer screenings. Based on the health belief model, other studies have demonstrated the relevance of psychological factors (e.g., subjective risk or use of cancer screenings, or subjective efficacy or fear) for cancer screenings (Adegboyega et al., 2022, Fawns-Ritchie et al., 2022, Hajek et al., 2021).

However, to date, only few studies have examined the association between personality factors and the use of cancer screenings (e.g. (Aschwanden et al., 2019, Gale et al., 2015)). The inclusion of personality factors has been suggested for consideration in the utilization of healthcare services (Hajek and König, 2020). In fact, a recent systematic review (Hajek et al., 2020) synthesized the published evidence and concluded that personality factors (especially in terms of higher conscientiousness and higher extraversion) are associated with an increased use of cancer screenings. That review also identified some gaps in knowledge. More precisely, it concluded that “future research is necessary to examine the link between personality factors and all sorts of cancer screening in further detail since the strength of the association between factors like conscientiousness and different sorts of cancer screenings may vary” (page 13) (Hajek et al., 2020). Therefore, the aim of this study was to examine the association between personality characteristics and use of different cancer screenings to address this gap in knowledge. Such knowledge may help to better understand non-participation in cancer screening examinations from a psychological perspective.

In terms of personality factors, five main traits can be distinguished (Digman, 1990): conscientiousness (tendency to be structured and planned), extraversion (tendency to be outgoing and energetic), agreeableness (tendency to be friendly and compassionate), neuroticism (tendency to be nervous and, more generally, experience negative emotions), and openness to experience (tendency to be curious and inventive).

## 2 Methods

### 2.1 Sample

For this study, data of the the German National Cohort (NAKO, “NAKO Gesundheitsstudie”) were used. NAKO is a large, multidisciplinary, population-based prospective cohort study. In sum, more than 205,000 women and men (19 to 74 years of age at recruitment) were recruited as random samples from 18 study centres in Germany between 2014 and 2019. It is currently the largest prospective cohort study in Germany. The baseline assessment covers a self-administered questionnaire, a face-to-face interview and several biomedical examinations – underlining the importance of the NAKO. The overall response rate in the baseline assessment equaled 17 % (from 9 % to 32 %, depending on the study centre). Further details are provided elsewhere (Peters et al., 2022).

Local ethics committees of all study centres approved the NAKO. It is conducted in accordance with the Declaration of Helsinki. Written informed consent was obtained from all participants included in the study.

### 2.2 Dependent variables

Self-reported information on previous use of cancer screening in the past five years was obtained by questionnaire at recruitment. The following cancer screening exams (reflecting the screenings offered in Germany at the time of recruitment) were covered and used as outcome variables in our analysis:

1. stool examination for blood (haemoccult test, early detection of bowel cancer) if age ≥50 years
2. colonoscopy (screening for colorectal cancer) if age ≥55 years
3. skin examination for moles (early detection of skin cancer) if age ≥35 years
4. breast palpation by a doctor (early detection of breast cancer) if women and age ≥30 years
5. x-ray examination of the breast (“mammography”, early detection of breast cancer) if women and age ≥50 years
6. cervical smear test if women
7. finger examination of the rectum (early detection of prostate cancer) if men and age ≥45 years
8. blood test for prostate cancer (determination of Prostate-Specific Antigen level (PSA) level) if men and age ≥45 years

In each case, the following answer options were given: “No”; “Yes, one time”; “Yes, several times”. We dichotomized all outcome measures (no; yes (including: “Yes, one time” and “Yes, several times”).

The analyses of the different types of screening untilization were based on the respective subsample of the NAKO cohort.

### 2.3 Key independent variables

The established Big Five Inventory-SOEP (BFI-S (Gerlitz and Schupp, 2005)) was used to quantify personality factors. It consists of 15 items (corresponding to three items per dimension). On a seven-point Likert scale which ranges from 1 = „does not apply to met at all“ to 7 = „applies to me perfectly“, each item was rated. By averaging the respective items, scores were generated for conscientiousness, extraversion, agreeableness, openness to experience and neuroticism. That tool has been documented to have satisfactory psychometric characteristics (Hahn et al., 2012).

### 2.4 Covariates

Our models were adjusted for several covariates based on the established Andersen model of healthcare utilization (Andersen, 1995). With regard to predisposing characteristics, we included the following variables in regression analysis: age (in years), sex (men; women), marital status (single; married, living together; married, living separated; divorced, widowed), education (pupil, attending a full-time general education school; left school without a secondary school leaving certificate/vocational school leaving certificate; lower secondary school leaving certificate/elementary school leaving certificate; secondary school leaving certificate/middle school leaving certificate; polytechnic secondary school of the GDR (German Democratic Republic) with completion of the 8th or 9th grade; polytechnic secondary school of the GDR with completion of the 10th grade; advanced technical college entrance qualification, completion of a specialized secondary school; general or subject-linked higher education entrance qualification/baccalaureate, grammar school or EOS (Extended Secondary School), also EOS with apprenticeship; school-leaving certificate obtained via a second educational pathway; another school-leaving qualification), and employment status (full-time employed; part-time employed; semi-retirement; marginally employed, 450 Euro or mini-job; one-euro job; occasionally or irregularly employed; in vocational training/apprenticeship; in retraining; federal voluntary service, voluntary social/ecological year; maternity, parental leave, parental leave or other leave of absence; not gainfully employed [including: pupils or students not working for money, unemployed, early retirees, pensioners without additional income]. With regard to enabling resources, we included the following factors in regression analysis: number of close friends (from 0 to 10 (truncated)) and (log) monthly net equivalent income in Euro.

With regard to need factors, we included the following in regression analysis: self-rated health (from 1 = poor to 5 = excellent; single-item measure), number of chronic conditions (count score of 45 chronic conditions (in each case: 0 = absence, 1 = presence): heart attack; narrowing of the coronary arteries or angina pectoris; heart failure or cardiac insufficiency; cardiac arrhythmia; shopfloor disease or circulatory disorders in the legs, also known as intermittent claudication or arterial occlusive disease; high blood pressure; cancer; diabetes or diabetes mellitus; elevated blood lipids or cholesterol or triglycerides; gout or uric acid disease; thyroid disease; back pain for 3 months or longer, almost every day; osteoporosis; osteoarthritis or joint wear and tear; stomach ulcer or duodenal ulcer; heartburn or reflux of stomach acid into the esophagus; ulcerative colitis or Crohn's disease; gallstones; cirrhosis of the liver; neurodermatitis or atopic eczema; Psoriasis; restricted kidney function or chronic renal insufficiency; stroke; seizure or epileptic seizure; migraine; Parkinson's syndrome, also called shaking palsy; depression; anxiety disorder or panic attack; cataract; glaucoma; macular degeneration; tuberculosis; shingles; HIV (Human Immunodeficiency Virus) infection or AIDS (Acquired Immunodeficiency Syndrome) disease; hepatitis B; hepatitis C; rheumatoid arthritis/polyarthritis; Bekhterev's disease/ankylosing spondylitis; systemic lupus erythematosus; Sjögren's syndrome; fibromyalgia; stones in the kidney, ureter or bladder; inflammation of one/both optic nerves; multiple sclerosis; tinnitus).

### 2.5 Statistical analysis

In a first step, we characterized each subsample eligible for the respective screening examination – stratified by sex if applicable. Then, sample characteristics for the maximal analytical sample (with skin examination for moles as outcome measure) are shown – also stratified by sex. Thereafter, unadjusted and adjusted logistic regressions were conducted to examine the association between personality factors and the likelihood of cancer screenings – partially restricted to men or women (or certain age groups), depending on the outcome used (see the section dependent variables and Table 1 for further details regarding the restrictions). Personality characteristics were entered simultaneously in the regression (in a sensitivity analysis, they were entered separately). We also estimated models with standardized personality factors.

**Table 1:** Sample sizes for the screening procedures (in parentheses: sample sizes for the analytical samples; Germany, between 2014 and 2019).

Screening procedure	Relevant age range and sex	N_males_	N_females_	N_total_
Stool examination for blood (haemoccult test, early detection of bowel cancer)	≥50 years, both sexes	48,730 (40,621)	48,740 (37,828)	97,470 (78,449)
Colonoscopy (screening for colorectal cancer)	≥55 years, both sexes	35,982 (29,510)	35,506 (26,814)	71,488 (56,324)
Skin examination for moles (early detection of skin cancer)	≥ 35 years, both sexes	79,079 (67,781)	79,669 (64,517)	158,748 (132,298)
Breast palpation by a doctor (early detection of breast cancer)	≥30 years, women	–	85,483 (69,804)	85,483 (69,804)
X-ray examination of the breast (“mammography”, early detection of breast cancer)	≥50 years, women	–	49,754 (38,571)	49,754 (38,571)
Cervical smear test	No age restriction, women	–	92,426 (76,124)	92,426 (76,124)
Finger examination of the rectum (early detection of prostate cancer)	≥45 years, men	64,149 (54,268)	–	64,149 (54,268)
Blood test for prostate cancer (determination of PSA level)	≥45 years, men	59,695 (50,448)	–	59,695 (50,448)

The respective sample size includes the relevant age and gender and refers only to persons who have provided information on the respective screening examination.

For large samples, the p-values can be significant (e.g., lower than 0.05) for the variables of interest even though the differences may be tiny. Therefore, following recent work by Connolly et al. (Connolly et al., 2022), we additionally used thresholds for adjusted odds ratios (aOR) at 1.43 or 0.70 to indicate *practical significance*. This corresponds to Cohen’s d values of ≥0.2 (a small effect size) following Chinn’s procedure to convert odds ratios to Cohen’s d (Chinn, 2000). All variables contained some missing values. We performed a complete case analysis. Thus, listwise deletion was used to handle missings. We also reported Cohen’s d to facilitate a clearer understanding of the relative associations. Stata 16.1 (StataCorp, College Station, TX, USA) was used for performing statistical analyses.

## 3 Results

### 3.1 Sample characteristics

Since the screening procedures differed with respect to sex and age range, the relevant sample sizes for the analysis differ considerably. Table 1 gives the characteristics and the figures.

Sample characteristics for the maximal analytical sample (here: for the adjusted logistic regression with skin examination for moles as outcome) by sex are shown in Table 2. It may be worth noting: The proportions (as well as means and SDs) are mostly very similar in the other analytical samples and are thus not presented here (but available upon request from the authors).

**Table 2:** Sample characteristics for the analytical sample – also stratified by sex (with skin examination for moles as outcome measure; n = 132,298; Germany, between 2014 and 2019).

Variables	Total sample (35 years and over)	Men (35 years and over)	Women (35 years and over)	p-values
	Mean (SD) /n (%)	Mean (SD) /n (%)	Mean (SD) /n (%)	
Age (in years)	53.0 (9.2)	53.2 (9.3)	52.8 (9.1)	<0.001
Sex				
Men	67,781 (51.2)	67,781 (100.0)	0 (0.0)	
Women	64,517 (48.8)	0 (0.0)	64,517 (100.0)	
Marital status				<0.001
Single	24,248 (18.3)	12,769 (18.8)	11,479 (17.8)	
Married, living together	85,627 (64.7)	46,268 (68.3)	39,359 (61.0)	
Married, living separated	2675 (2.0)	1268 (1.9)	1407 (2.2)	
Divorced	15,998 (12.1)	6577 (9.7)	9421 (14.6)	
Widowed	3750 (2.8)	899 (1.3)	2851 (4.4)	
Education				<0.001
Pupil, attending a full-time general education school	31 (0.0)	18 (0.0)	13 (0.0)	
Left school without a secondary school leaving certificate/vocational school leaving certificate	1112 (0.8)	637 (0.9)	475 (0.7)	
Lower secondary school leaving certificate/elementary school leaving certificate	15,675 (11.8)	8949 (13.2)	6726 (10.4)	
Secondary school leaving certificate/middle school leaving certificate	24,808 (18.8)	10,663 (15.7)	14,145 (21.9)	
Polytechnic secondary school of the GDR with completion of the 8th or 9th grade	1323 (1.0)	863 (1.3)	460 (0.7)	
Polytechnic secondary school of the GDR with completion of the 10th grade	18,923 (14.3)	8976 (13.2)	9947 (15.4)	
Advanced technical college entrance qualification, completion of a specialized secondary school	13,771 (10.4)	7942 (11.7)	5829 (9.0)	
General or subject-linked higher education entrance qualification/baccalaureate, grammar school or EOS, also EOS with apprenticeship	52,536 (39.7)	27,412 (40.4)	25,124 (38.9)	
School-leaving certificate obtained via a second educational pathway	3333 (2.5)	1890 (2.8)	1443 (2.2)	
Another school-leaving qualification	786 (0.6)	431 (0.6)	355 (0.6)	
Employment status				<0.001
Full-time employed	71,393 (54.0)	46,846 (69.1)	24,547 (38.0)	
Part-time employed	24,665 (18.6)	3927 (5.8)	20,738 (32.1)	
Semi-retirement	1343 (1.0)	745 (1.1)	598 (0.9)	
Marginally employed, 450 Euro or mini-job	4617 (3.5)	1636 (2.4)	2981 (4.6)	
One-euro job	95 (0.1)	59 (0.1)	36 (0.1)	
Occasionally or irregularly employed	891 (0.7)	486 (0.7)	405 (0.6)	
In vocational training/apprenticeship	78 (0.1)	28 (0.0)	50 (0.1)	
In retraining	144 (0.1)	77 (0.1)	67 (0.1)	
Federal voluntary service, voluntary social/ecological year	19 (0.0)	9 (0.0)	10 (0.0)	
Maternity, parental leave, parental leave or other leave of absence	867 (0.7)	173 (0.3)	694 (1.1)	
Not gainfully employed	28,186 (21.3)	13,795 (20.4)	14,391 (22.3)	
Monthly net equivalent income (in Euro)	2446.5 (1529.1)	2590.8 (1662.4)	2294.9 (1358.9)	<0.001
Number of close friends	3.5 (2.4)	3.4 (2.5)	3.7 (2.4)	<0.001
Number of chronic conditions	3.0 (2.5)	2.8 (2.4)	3.3 (2.6)	<0.001
Self-rated health	3.2 (0.7)	3.2 (0.7)	3.2 (0.7)	<0.001
Conscientiousness	5.8 (0.9)	5.7 (1.0)	5.9 (0.9)	<0.001
Extraversion	4.7 (1.2)	4.6 (1.2)	4.8 (1.2)	<0.001
Agreeableness	5.6 (1.0)	5.5 (1.0)	5.7 (1.0)	<0.001
Openness to experience	4.6 (1.3)	4.6 (1.2)	4.6 (1.3)	<0.001
Neuroticism	3.4 (1.4)	3.2 (1.3)	3.7 (1.4)	<0.001

Notes: P-values are based on *t*-tests or Chi^2^-tests, as appropriate.

In the displayed analytical sample, mean age equaled 53.0 years (SD: 9.2 years), with 48.8 % of the individuals being female. About 64.7 % of the individuals were married and living together with their spouse. In sum, 39.7 % of the individuals had a general or subject-linked higher education entrance qualification and 54.0 % of the individuals were full-time employed. On average, individuals had 3.0 chronic conditions (SD: 2.5).

**Fig. 1:**
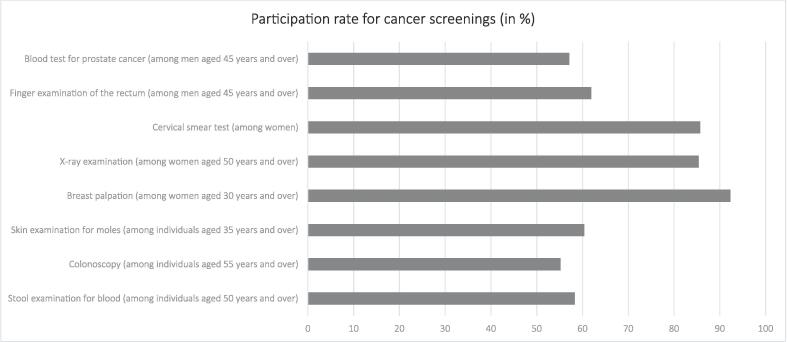
Participation rate for cancer screenings (in %; Germany, between 2014 and 2019).

With regard to personality factors, average conscientiousness score was 5.8 (SD: 0.9), average extraversion score was 4.7 (SD: 1.2), average agreeableness score was 5.6 (SD: 1.0), average openness to experience score was 4.6 (SD: 1.3) and average neuroticism score was 3.4 (SD: 1.4). Please see Table 2 for further details regarding the sample characteristics in general. The participation rate for the cancer screenings are shown in Fig. 1.

### 3.2 Regression analysis

Results of unadjusted logistic regressions (for the association between personality characteristics and cancer screenings) are shown in Supplementary Table 1. Additionally, results of adjusted logistic regressions are given in Table 3 (the adjusted model with covariates displayed is shown in Supplementary Table 2). Higher odds of, for example, skin examination for moles was associated with higher conscientiousness (OR: 1.07, p < 0.001), higher extraversion (OR: 1.03, p < 0.001), higher agreeableness (OR: 1.02, p < 0.001), lower openness to experience (OR: 0.98, p < 0.001) and higher neuroticism (OR: 1.07, p < 0.001) among the total sample. Depending on the outcome used, the associations slightly varied. In Supplementary Table 3, odds ratios (with standardized personality factors) are reported. These results were comparable with the results shown in Table 3. In Supplementary Table 4, the effect sizes (in terms of Cohen’s d) were displayed (to get a better picture). The conventional interpretation is (Cohen, 2013): |d| = 0.20 (small), |d| = 0.50 (medium) and |d|=0.80 (large). Even though all effect sizes were smaller than 0.20, particularly the associations between the three highlighted personality factors (i.e., conscientiousness, neuroticism and extraversion) and the likelihood of breast palpation by a doctor seem worth noting. In Supplementary Tables 5 to 12, adjusted logistic regressions are displayed where personality characteristics were entered separately.

**Table 3:** Personality and likelihood of cancer screening. Results of adjusted logistic regressions (Germany, between 2014 and 2019).

Independent variables	Outcomes							
	Stool examination for blood (haemoccult test, early detection of bowel cancer) - among the total sample	Colonoscopy (screening for colorectal cancer) - among the total sample	Skin examination for moles (early detection of skin cancer) - among the total sample	Breast palpation by a doctor (early detection of breast cancer) - only among women	X-ray examination of the breast (“mammography”, early detection of breast cancer) - only among women	Cervical smear test - only among women	Finger examination of the rectum (early detection of prostate cancer) - only among men	Blood test for prostate cancer (determination of PSA level) - only among men
Conscientiousness	1.08***	1.04***	1.07***	1.18***	1.15***	1.13***	1.07***	1.10***
	(1.06–1.10)	(1.02–1.06)	(1.05–1.08)	(1.14–1.22)	(1.11–1.19)	(1.10–1.15)	(1.05–1.09)	(1.08–1.12)
Extraversion	1.03***	1.07***	1.03***	1.12***	1.06***	1.07***	1.05***	1.07***
	(1.02–1.04)	(1.05–1.09)	(1.02–1.05)	(1.09–1.15)	(1.03–1.09)	(1.05–1.09)	(1.03–1.07)	(1.05–1.09)
Agreeableness	1.02*	1.01	1.02***	1.09***	1.07***	1.05***	1.04***	1.04***
	(1.00–1.03)	(0.99–1.03)	(1.01–1.04)	(1.05–1.12)	(1.04–1.11)	(1.03–1.08)	(1.02–1.07)	(1.01–1.06)
Openness to experience	1.00	0.96***	0.98***	0.95***	0.88***	0.98*	0.98*	0.97***
	(0.98–1.01)	(0.95–0.98)	(0.97–0.99)	(0.93–0.97)	(0.85–0.90)	(0.96–1.00)	(0.96–1.00)	(0.95–0.99)
Neuroticism	1.06***	1.08***	1.07***	1.16***	1.09***	1.13***	1.10***	1.10***
	(1.05–1.07)	(1.07–1.10)	(1.06–1.08)	(1.14–1.19)	(1.06–1.11)	(1.11–1.15)	(1.08–1.12)	(1.08–1.12)
Covariates	✓	✓	✓	✓	✓	✓	✓	✓
Pseudo R^2^	0.025	0.034	0.033	0.053	0.053	0.080	0.087	0.101
Observations	78,449	56,324	132,298	69,804	38,571	76,124	54,268	50,448

Notes: Odds Ratios are displayed, 95 % CI in parentheses, *** p < 0.001, ** p < 0.01, * p < 0.05, + p < 0.10; findings of practical importance (OR: ≥1.43 or ≤0.70) would be in bold.

Adjusted for age, sex (if applicable), marital status, education, employment status, study center, income, number of close friends, number of chronic conditions, and self-rated health.

## 4 Discussion

Utilizing data from the NAKO, our study aim was to examine the association between personality factors and the utilization of various cancer screenings (self-reported). In regression analysis, particularly higher conscientiousness, and higher neuroticism (and higher extraversion) were associated with a higher likelihood of using different cancer screenings. However, the practical significance of these associations appears limited, as indicated by the effect sizes. The differences in effect sizes based on the type of cancer screening used as the outcome were found to be relatively minor.

This current study extends our current knowledge by examining a *wide array of cancer screening procedures* – and is therefore not restricted to single cancer screenings (Arai et al., 2009, Gale et al., 2015, Hill and Gick, 2013, Pandhi et al., 2016, Schwartz et al., 1999, Sen and Kumkale, 2016). In fact, only a few studies examined the association between personality characteristics and several cancer screening procedures (e.g., (Aschwanden et al., 2019, Nolan et al., 2019)).

Most of the previous studies also found a link between higher conscientiousness and a higher likelihood of using cancer screenings (Aschwanden et al., 2019, Nolan et al., 2019, Pandhi et al., 2016, Schwartz et al., 1999, Sen and Kumkale, 2016). It should be noted that previous studies mostly found an association between higher conscientiousness and a higher likelihood of mammography (e.g., (Pandhi et al., 2016, Sen and Kumkale, 2016)). Given the fact that individuals scoring high in conscientiousness tend to be forward-planners, rule-followers, and goal- and task-oriented, the establishment of such a link appears easily understandable. Such individuals are thus likely to follow the recommendations for cancer screenings (Hajek et al., 2020).

In our study, higher neuroticism was associated with a higher likelihood of using cancer screenings. This adds to the inconclusive (mostly non-significant) results identified thus far (Hajek et al., 2020). We assume that particularly fears or worries about diseases may drive the higher probability of using cancer screenings among individuals with high levels of neuroticism (Hajek et al., 2020). This may overcompensate for possible fears of illness, which in turn could lead to avoidance or denial behavior (e.g. avoiding cancer screenings).

Regarding the existing evidence with many non-significant associations: It is possible that this avoidance behavior was more pronounced in other populations and compensated for possible effects (in the sense of: more anxiety leads to more screenings) - which could explain the many non-significant results in the literature.

Also, our study showed an association between higher extraversion and a higher likelihood of using cancer screenings. This is supported by a previous systematic review (Hajek et al., 2020). One explanation may be that higher extraversion is linked to a higher level of positive emotions. Positive emotions are in turn associated with positive expectations (Arampatzi et al., 2020, Taylor et al., 2000). Individuals scoring high in extraversion might have more positive expectations with regard to several cancer screening procedures (Aschwanden et al., 2019).

The association between a higher level of agreeableness and a higher likelihood of (most) cancer screenings can be explained by the fact that individuals scoring high in agreeableness may prefer to avoid disagreements with doctors when making decisions (Sen and Kumkale, 2016).

Initially, we expected that individuals who score high in openness to experience have a *higher* likelihood of using cancer screenings because openness reflects general open mindedness. A speculative explanation for the opposite association may be that individuals scoring high in openness to experience want to enjoy their life to the fullest (e.g. traveling) - and would therefore possibly be more likely to avoid cancer screenings because of the potential cancer diagnosis at screening. However, empirical evidence for such an explanation is lacking thus far and future research is therefore needed to confirm that notion.

Regarding the healthcare system in Germany: It should also be noted that access to healthcare (including cancer screenings) in Germany is quite good. For example, in Germany there is a free access to General Practitioners (GP) and specialists. Moreover, waiting times for such appointments are rather short (Zok, 2007). The access to healthcare could explain some differences between our results and former studies (which used data from countries with different access to healthcare such as the United States). Thus, we recommend future research regarding the association between personality factors and the use of cancer screenings in other countries with different access to health care.

Certain strengths and limitations are worth noting. Data were taken from a very large sample from 18 different regions from Germany. Although potential participants were randomly selected from population registries, more health conscious participants are likely to be overrepresented among those who actually participated. Reported use of the various screening tests is therefore most likely higher than the national average. Furthermore, no distinction was made between frequency of use of these tests. As common in such large studies, a brief, established tool was used to quantify personality factors. Various cancer screening procedures were included as outcome measures. Analyses were adjusted for several covariates that were selected based on the established Andersen model. Furthermore, covariates such as chronic conditions were assessed in detail. Due to the low response rate, the generalizability may be restricted, particularly for younger individuals (Schipf et al., 2020). It is important to be aware that this is a cross-sectional study with inherent limitations regarding directionality.

In conclusion, our study particularly showed an association between elevated levels of conscientiousness, neuroticism, and extraversion and an increased likelihood of utilizing various cancer screenings. Such knowledge may assist in characterizing individuals with a higher risk of underutilizing cancer screening services. Future longitudinal studies are clearly needed in this neglected research area.

## Declaration of competing interest

The authors declare that they have no known competing financial interests or personal relationships that could have appeared to influence the work reported in this paper.

## Data Availability

The authors do not have permission to share data.
