# Supplementary material for: Personality and the use of cancer screenings - Results of the German National Cohort
Source: Prev Med Rep. 2024 Mar 8;41:102677. doi: 10.1016/j.pmedr.2024.102677 (PMC10963220; doi:10.1016/j.pmedr.2024.102677)
Supplement: Supplementary data 1 [file mmc1.docx]

Supplementary Table 1. Personality and likelihood of cancer screening. Results of unadjusted logistic regressions (Germany, between 2014 and 2019)

| Outcomes  Independent variables | Stool examination for blood (haemoccult test, early detection of bowel cancer) - among the total sample | Colonoscopy (screening for colorectal cancer) - among the total sample | Skin examination for moles (early detection of skin cancer) - among the total sample | Breast palpation by a doctor (early detection of breast cancer)  - only among women | X-ray examination of the breast ("mammography", early detection of breast cancer) - only among women | Cervical smear test - only among women | Finger examination of the rectum (early detection of prostate cancer) - only among men | Blood test for prostate cancer (determination of PSA level) - only among men |
| --- | --- | --- | --- | --- | --- | --- | --- | --- |
| Conscientiousness | 1.07*** | 1.02+ | 1.07*** | 1.24*** | 1.18*** | 1.16*** | 1.06*** | 1.09*** |
|  | (1.05 - 1.08) | (1.00 - 1.03) | (1.05 - 1.08) | (1.20 - 1.27) | (1.14 - 1.21) | (1.13 - 1.18) | (1.05 - 1.08) | (1.07 - 1.11) |
| Extraversion | 1.03*** | 1.07*** | 1.06*** | 1.19*** | 1.07*** | 1.14*** | 1.03*** | 1.05*** |
|  | (1.02 - 1.04) | (1.05 - 1.08) | (1.05 - 1.07) | (1.17 - 1.22) | (1.05 - 1.10) | (1.12 - 1.16) | (1.02 - 1.05) | (1.04 - 1.07) |
| Agreeableness | 1.02** | 1.00 | 1.05*** | 1.10*** | 1.12*** | 1.06*** | 1.03*** | 1.02* |
|  | (1.01 - 1.03) | (0.98 - 1.02) | (1.03 - 1.06) | (1.07 - 1.13) | (1.09 - 1.15) | (1.04 - 1.08) | (1.02 - 1.05) | (1.01 - 1.04) |
| Openness to experience | 1.02** | 0.99+ | 1.01+ | 0.97** | 0.86*** | 1.01 | 1.00 | 1.01 |
|  | (1.00 - 1.03) | (0.98 - 1.00) | (1.00 - 1.02) | (0.95 - 0.99) | (0.84 - 0.87) | (0.99 - 1.03) | (0.99 - 1.02) | (0.99 - 1.02) |
| Neuroticism | 1.06*** | 1.08*** | 1.08*** | 1.15*** | 1.09*** | 1.12*** | 1.07*** | 1.04*** |
|  | (1.05 - 1.07) | (1.07 - 1.09) | (1.07 - 1.09) | (1.13 - 1.17) | (1.07 - 1.11) | (1.11 - 1.14) | (1.06 - 1.08) | (1.02 - 1.05) |
| Pseudo R² | 0.002 | 0.003 | 0.004 | 0.017 | 0.011 | 0.011 | 0.002 | 0.003 |
| Observations | 93,663 | 68,239 | 154,087 | 82,715 | 47,480 | 89,700 | 62,366 | 58,010 |

Notes: Odds Ratios are displayed, 95% CI in parentheses, *** p<0.001, ** p<0.01, * p<0.05, + p<0.10

Supplementary Table 2. Personality and likelihood of cancer screening. Results of adjusted logistic regressions – with covariates displayed (Germany, between 2014 and 2019)

| Outcomes  Independent variables | Stool examination for blood (haemoccult test, early detection of bowel cancer) - among the total sample | Colonoscopy (screening for colorectal cancer) - among the total sample | Skin examination for moles (early detection of skin cancer) - among the total sample | Breast palpation by a doctor (early detection of breast cancer)  - only among women | X-ray examination of the breast ("mammography", early detection of breast cancer) - only among women | Cervical smear test - only among women | Finger examination of the rectum (early detection of prostate cancer) - only among men | Blood test for prostate cancer (determination of PSA level) - only among men |
| --- | --- | --- | --- | --- | --- | --- | --- | --- |
| Key independent variables: |  |  |  |  |  |  |  |  |
| Conscientiousness | 1.08*** | 1.04*** | 1.07*** | 1.18*** | 1.15*** | 1.13*** | 1.07*** | 1.10*** |
|  | (1.06 - 1.10) | (1.02 - 1.06) | (1.05 - 1.08) | (1.14 - 1.22) | (1.11 - 1.19) | (1.10 - 1.15) | (1.05 - 1.09) | (1.08 - 1.12) |
| Extraversion | 1.03*** | 1.07*** | 1.03*** | 1.12*** | 1.06*** | 1.07*** | 1.05*** | 1.07*** |
|  | (1.02 - 1.04) | (1.05 - 1.09) | (1.02 - 1.05) | (1.09 - 1.15) | (1.03 - 1.09) | (1.05 - 1.09) | (1.03 - 1.07) | (1.05 - 1.09) |
| Agreeableness | 1.02* | 1.01 | 1.02*** | 1.09*** | 1.07*** | 1.05*** | 1.04*** | 1.04*** |
|  | (1.00 - 1.03) | (0.99 - 1.03) | (1.01 - 1.04) | (1.05 - 1.12) | (1.04 - 1.11) | (1.03 - 1.08) | (1.02 - 1.07) | (1.01 - 1.06) |
| Openness to experience | 1.00 | 0.96*** | 0.98*** | 0.95*** | 0.88*** | 0.98* | 0.98* | 0.97*** |
|  | (0.98 - 1.01) | (0.95 - 0.98) | (0.97 - 0.99) | (0.93 - 0.97) | (0.85 - 0.90) | (0.96 - 1.00) | (0.96 - 1.00) | (0.95 - 0.99) |
| Neuroticism | 1.06*** | 1.08*** | 1.07*** | 1.16*** | 1.09*** | 1.13*** | 1.10*** | 1.10*** |
|  | (1.05 - 1.07) | (1.07 - 1.10) | (1.06 - 1.08) | (1.14 - 1.19) | (1.06 - 1.11) | (1.11 - 1.15) | (1.08 - 1.12) | (1.08 - 1.12) |
|  |  |  |  |  |  |  |  |  |
| Covariates: |  |  |  |  |  |  |  |  |
|  |  |  |  |  |  |  |  |  |
|  |  |  |  |  |  |  |  |  |
| Age (in years) | 1.03*** | 1.05*** | 1.01*** | 0.98*** | 1.06*** | 0.96*** | 1.08*** | 1.09*** |
|  | (1.03 - 1.04) | (1.04 - 1.05) | (1.00 - 1.01) | (0.97 - 0.98) | (1.06 - 1.07) | (0.95 - 0.96) | (1.08 - 1.08) | (1.08 - 1.09) |
| Sex: Women (Reference category: Men) | 0.98 | 0.85*** | 1.28*** |  |  |  |  |  |
|  | (0.94 - 1.01) | (0.82 - 0.88) | (1.24 - 1.31) |  |  |  |  |  |
| Marital status: - Married, living together (Reference category: Single) | 1.25*** | 1.29*** | 1.13*** | 1.81*** | 1.62*** | 1.70*** | 1.40*** | 1.43*** |
|  | (1.20 - 1.31) | (1.22 - 1.37) | (1.10 - 1.17) | (1.68 - 1.96) | (1.48 - 1.77) | (1.60 - 1.81) | (1.32 - 1.47) | (1.35 - 1.51) |
| - Married, living separated | 1.03 | 1.16* | 0.94 | 1.67*** | 1.14 | 1.46*** | 1.30*** | 1.17* |
|  | (0.92 - 1.15) | (1.01 - 1.33) | (0.87 - 1.03) | (1.35 - 2.06) | (0.94 - 1.39) | (1.25 - 1.71) | (1.14 - 1.50) | (1.01 - 1.35) |
| - Divorced | 1.02 | 1.11** | 0.97 | 1.55*** | 1.09+ | 1.47*** | 1.21*** | 1.17*** |
|  | (0.96 - 1.08) | (1.03 - 1.19) | (0.93 - 1.01) | (1.40 - 1.71) | (0.99 - 1.20) | (1.36 - 1.58) | (1.13 - 1.30) | (1.09 - 1.27) |
| - Widowed | 1.10* | 1.13* | 0.91* | 1.53*** | 1.37*** | 1.37*** | 1.22* | 1.42*** |
|  | (1.01 - 1.19) | (1.03 - 1.24) | (0.85 - 0.98) | (1.32 - 1.77) | (1.19 - 1.58) | (1.22 - 1.52) | (1.04 - 1.43) | (1.20 - 1.67) |
| Education: - Pupil, attending a full-time generation education school (Reference category: general or subject-linked higher education entrance qualification/baccalaureate, grammar school or EOS, also EOS with apprenticeship | 0.95 | 0.85 | 1.59 | 1.20 | 0.72 | 1.20 | 1.11 | 0.63 |
|  | (0.42 - 2.13) | (0.38 - 1.90) | (0.70 - 3.57) | (0.16 - 9.29) | (0.15 - 3.49) | (0.35 - 4.15) | (0.34 - 3.64) | (0.22 - 1.86) |
| - Left school without a secondary school leaving certificate/vocational school leaving certificate | 0.87* | 0.85* | 0.57*** | 0.60*** | 1.23 | 0.42*** | 0.75** | 0.72*** |
|  | (0.76 - 1.00) | (0.73 - 0.99) | (0.50 - 0.64) | (0.46 - 0.78) | (0.91 - 1.66) | (0.35 - 0.52) | (0.62 - 0.90) | (0.59 - 0.87) |
| - Lower secondary school leaving certificate/elementary school leaving certificate | 0.96+ | 1.02 | 0.73*** | 0.72*** | 1.41*** | 0.61*** | 0.91** | 0.80*** |
|  | (0.91 - 1.01) | (0.97 - 1.08) | (0.71 - 0.77) | (0.65 - 0.80) | (1.27 - 1.57) | (0.57 - 0.66) | (0.85 - 0.97) | (0.75 - 0.86) |
| - Secondary school leaving certificate/middle school leaving certificate | 1.02 | 1.02 | 0.91*** | 0.93+ | 1.34*** | 0.87*** | 1.03 | 0.93* |
|  | (0.97 - 1.06) | (0.97 - 1.08) | (0.88 - 0.94) | (0.86 - 1.01) | (1.24 - 1.46) | (0.82 - 0.93) | (0.97 - 1.10) | (0.88 - 0.99) |
| - Polytechnic secondary school of the GDR with completion of the 8th or 9th grade | 0.79*** | 1.05 | 0.61*** | 0.80 | 1.88** | 0.62*** | 0.85* | 0.80** |
|  | (0.70 - 0.90) | (0.91 - 1.21) | (0.55 - 0.69) | (0.59 - 1.09) | (1.23 - 2.89) | (0.50 - 0.78) | (0.72 - 1.00) | (0.67 - 0.94) |
| - Polytechnic secondary school of the GDR with completion of the 10th grade | 0.97 | 1.02 | 0.89*** | 1.22*** | 1.44*** | 0.95 | 0.97 | 0.90** |
|  | (0.92 - 1.03) | (0.96 - 1.09) | (0.85 - 0.93) | (1.09 - 1.37) | (1.29 - 1.61) | (0.87 - 1.03) | (0.91 - 1.04) | (0.84 - 0.96) |
| - Advanced technical college entrance qualification, completion of a specialized secondary school | 1.06* | 1.05 | 1.01 | 1.01 | 1.18** | 0.96 | 1.05 | 1.01 |
|  | (1.01 - 1.12) | (0.99 - 1.12) | (0.97 - 1.05) | (0.91 - 1.13) | (1.06 - 1.31) | (0.88 - 1.04) | (0.98 - 1.12) | (0.94 - 1.08) |
| - School-leaving certificate obtained via a second educational pathway | 0.99 | 1.00 | 0.92* | 0.87 | 0.92 | 0.95 | 0.98 | 0.89+ |
|  | (0.91 - 1.09) | (0.90 - 1.11) | (0.85 - 0.99) | (0.72 - 1.05) | (0.77 - 1.10) | (0.82 - 1.10) | (0.87 - 1.10) | (0.79 - 1.00) |
| - Another school-leaving qualification | 0.71*** | 0.99 | 0.52*** | 0.48*** | 1.25 | 0.44*** | 0.61*** | 0.79+ |
|  | (0.58 - 0.86) | (0.78 - 1.25) | (0.45 - 0.60) | (0.36 - 0.65) | (0.83 - 1.89) | (0.35 - 0.56) | (0.48 - 0.77) | (0.61 - 1.02) |
| Employment status: - Part-time employed (Reference category: Full-time employed) | 1.06** | 1.06* | 1.17*** | 1.25*** | 1.01 | 1.32*** | 0.96 | 0.97 |
|  | (1.02 - 1.11) | (1.00 - 1.13) | (1.13 - 1.21) | (1.16 - 1.36) | (0.94 - 1.09) | (1.24 - 1.40) | (0.88 - 1.04) | (0.89 - 1.06) |
| - Semi-retirement | 1.36*** | 1.30*** | 1.42*** | 1.02 | 1.09 | 1.04 | 1.08 | 1.18+ |
|  | (1.20 - 1.53) | (1.16 - 1.46) | (1.26 - 1.61) | (0.74 - 1.39) | (0.82 - 1.44) | (0.83 - 1.30) | (0.91 - 1.29) | (0.99 - 1.40) |
| - Marginally employed, 450 Euro or mini-job | 0.94+ | 1.10* | 1.00 | 1.01 | 0.97 | 0.93 | 0.85** | 0.88+ |
|  | (0.87 - 1.01) | (1.01 - 1.20) | (0.93 - 1.06) | (0.88 - 1.17) | (0.84 - 1.12) | (0.85 - 1.03) | (0.75 - 0.96) | (0.78 - 1.00) |
| - One-euro job | 0.72 | 1.19 | 0.62* | 0.51+ | 0.59 | 0.42* | 0.77 | 0.44* |
|  | (0.43 - 1.20) | (0.67 - 2.13) | (0.41 - 0.95) | (0.24 - 1.10) | (0.22 - 1.53) | (0.22 - 0.82) | (0.43 - 1.39) | (0.21 - 0.90) |
| - Occasionally or irregularly employed | 0.80** | 1.02 | 0.98 | 0.68** | 0.58*** | 0.81+ | 0.92 | 0.91 |
|  | (0.68 - 0.93) | (0.86 - 1.20) | (0.86 - 1.13) | (0.50 - 0.91) | (0.43 - 0.78) | (0.65 - 1.01) | (0.74 - 1.15) | (0.73 - 1.14) |
| - In vocational training/apprenticeship | 2.43 | 1.01 | 0.83 | 0.49* | 1.60 | 0.59** | 1.02 | 2.09 |
|  | (0.74 - 8.03) | (0.06 - 16.27) | (0.53 - 1.30) | (0.26 - 0.93) | (0.18 - 14.23) | (0.43 - 0.81) | (0.37 - 2.81) | (0.72 - 6.09) |
| - In retraining | 1.55 | 0.83 | 0.84 | 0.61 | 1.52 | 0.76 | 0.62 | 0.83 |
|  | (0.81 - 2.98) | (0.34 - 2.03) | (0.60 - 1.17) | (0.32 - 1.18) | (0.33 - 7.11) | (0.42 - 1.37) | (0.33 - 1.19) | (0.42 - 1.63) |
| - Federal voluntary service, voluntary social/ecological year | 1.38 | 0.84 | 0.62 | 1.12 | - | 0.40+ | 2.52 | 1.86 |
|  | (0.44 - 4.31) | (0.22 - 3.24) | (0.24 - 1.60) | (0.14 - 8.89) |  | (0.14 - 1.18) | (0.47 - 13.70) | (0.40 - 8.67) |
| - Maternity, parental leave, parental leave or other leave of absence | 0.75 | 1.36 | 0.93 | 1.45* | 1.42 | 2.48*** | 1.65+ | 1.54 |
|  | (0.47 - 1.21) | (0.75 - 2.48) | (0.81 - 1.07) | (1.09 - 1.94) | (0.55 - 3.64) | (1.85 - 3.32) | (0.92 - 2.99) | (0.85 - 2.78) |
| - Not gainfully employed | 0.89*** | 1.07** | 1.09*** | 0.85*** | 0.76*** | 0.86*** | 0.86*** | 0.87*** |
|  | (0.85 - 0.93) | (1.02 - 1.13) | (1.05 - 1.13) | (0.78 - 0.93) | (0.69 - 0.83) | (0.81 - 0.92) | (0.81 - 0.91) | (0.81 - 0.92) |
| Log monthly net equivalent income (in Euro) | 1.15*** | 1.32*** | 1.34*** | 1.54*** | 1.19*** | 1.52*** | 1.29*** | 1.62*** |
|  | (1.12 - 1.19) | (1.27 - 1.37) | (1.31 - 1.37) | (1.45 - 1.64) | (1.12 - 1.27) | (1.45 - 1.60) | (1.24 - 1.34) | (1.56 - 1.69) |
| Number of close friends (from 0 to 10) | 1.02*** | 1.03*** | 1.02*** | 1.08*** | 1.04*** | 1.05*** | 1.01* | 1.02*** |
|  | (1.01 - 1.03) | (1.02 - 1.03) | (1.02 - 1.03) | (1.07 - 1.10) | (1.03 - 1.05) | (1.04 - 1.06) | (1.00 - 1.02) | (1.01 - 1.03) |
| Study center: - Regensburg (Reference category: Augsburg) | 1.46*** | 1.33*** | 1.08* | 1.12 | 1.28** | 1.12+ | 1.27*** | 0.91+ |
|  | (1.34 - 1.58) | (1.21 - 1.46) | (1.02 - 1.15) | (0.96 - 1.30) | (1.09 - 1.49) | (1.00 - 1.26) | (1.15 - 1.41) | (0.82 - 1.01) |
| - Mannheim | 1.22*** | 1.23*** | 1.03 | 1.23** | 1.28** | 1.03 | 1.28*** | 1.17** |
|  | (1.12 - 1.32) | (1.12 - 1.35) | (0.97 - 1.10) | (1.06 - 1.43) | (1.10 - 1.49) | (0.92 - 1.16) | (1.16 - 1.42) | (1.05 - 1.29) |
| - Freiburg | 1.31*** | 1.38*** | 1.09** | 1.29** | 1.03 | 1.28*** | 1.20*** | 0.98 |
|  | (1.21 - 1.42) | (1.25 - 1.51) | (1.03 - 1.16) | (1.10 - 1.51) | (0.89 - 1.19) | (1.13 - 1.45) | (1.09 - 1.32) | (0.88 - 1.08) |
| - Saarbrücken | 1.16*** | 1.30*** | 1.14*** | 1.28** | 1.21* | 1.12+ | 1.29*** | 1.15* |
|  | (1.06 - 1.26) | (1.18 - 1.44) | (1.06 - 1.21) | (1.08 - 1.51) | (1.03 - 1.42) | (0.99 - 1.27) | (1.16 - 1.44) | (1.03 - 1.28) |
| - Essen | 1.22*** | 1.02 | 1.13*** | 1.17* | 1.41*** | 1.01 | 1.11* | 0.99 |
|  | (1.13 - 1.32) | (0.93 - 1.12) | (1.06 - 1.20) | (1.01 - 1.36) | (1.21 - 1.65) | (0.90 - 1.13) | (1.00 - 1.22) | (0.89 - 1.10) |
| - Münster | 1.33*** | 1.62*** | 1.19*** | 1.52*** | 1.69*** | 1.27*** | 1.64*** | 1.52*** |
|  | (1.22 - 1.44) | (1.47 - 1.78) | (1.12 - 1.27) | (1.28 - 1.80) | (1.44 - 1.98) | (1.13 - 1.44) | (1.48 - 1.82) | (1.37 - 1.69) |
| - Düsseldorf | 1.22*** | 1.28*** | 1.27*** | 1.40*** | 1.62*** | 1.30*** | 1.27*** | 1.22*** |
|  | (1.12 - 1.33) | (1.15 - 1.41) | (1.18 - 1.36) | (1.18 - 1.67) | (1.36 - 1.92) | (1.14 - 1.49) | (1.13 - 1.41) | (1.09 - 1.37) |
| - Halle | 0.76*** | 0.96 | 1.07* | 1.28** | 1.48*** | 1.26*** | 0.77*** | 0.87* |
|  | (0.70 - 0.83) | (0.87 - 1.06) | (1.00 - 1.15) | (1.08 - 1.52) | (1.25 - 1.76) | (1.11 - 1.43) | (0.69 - 0.86) | (0.78 - 0.97) |
| - Leipzig | 0.99 | 1.01 | 1.19*** | 1.43*** | 2.30*** | 1.36*** | 0.94 | 0.86** |
|  | (0.92 - 1.08) | (0.92 - 1.12) | (1.12 - 1.27) | (1.21 - 1.68) | (1.92 - 2.76) | (1.20 - 1.53) | (0.85 - 1.05) | (0.77 - 0.96) |
| - North Berlin | 0.86*** | 1.17** | 0.85*** | 1.16+ | 1.65*** | 1.17* | 1.01 | 0.89* |
|  | (0.80 - 0.94) | (1.06 - 1.29) | (0.80 - 0.91) | (0.99 - 1.37) | (1.39 - 1.95) | (1.03 - 1.32) | (0.91 - 1.13) | (0.80 - 0.99) |
| - Central Berlin | 0.91* | 1.10* | 0.87*** | 1.10 | 1.38*** | 1.11+ | 1.03 | 0.86** |
|  | (0.85 - 0.99) | (1.01 - 1.21) | (0.82 - 0.93) | (0.95 - 1.27) | (1.19 - 1.60) | (0.99 - 1.24) | (0.93 - 1.13) | (0.78 - 0.96) |
| - South Berlin | 0.91* | 1.19*** | 1.06+ | 1.40*** | 1.36*** | 1.38*** | 1.10+ | 0.88* |
|  | (0.84 - 0.98) | (1.08 - 1.31) | (1.00 - 1.13) | (1.19 - 1.64) | (1.17 - 1.59) | (1.22 - 1.56) | (0.99 - 1.22) | (0.79 - 0.98) |
| - Hannover | 1.23*** | 1.15** | 1.17*** | 1.41*** | 1.53*** | 1.17* | 1.39*** | 1.23*** |
|  | (1.13 - 1.33) | (1.05 - 1.27) | (1.10 - 1.25) | (1.19 - 1.65) | (1.31 - 1.80) | (1.03 - 1.32) | (1.26 - 1.55) | (1.10 - 1.37) |
| - Hamburg | 0.98 | 1.32*** | 1.25*** | 1.30*** | 1.75*** | 1.11+ | 1.26*** | 0.97 |
|  | (0.91 - 1.06) | (1.20 - 1.45) | (1.17 - 1.33) | (1.12 - 1.52) | (1.50 - 2.05) | (0.99 - 1.24) | (1.14 - 1.39) | (0.87 - 1.07) |
| - Bremen | 1.51*** | 1.29*** | 1.22*** | 1.38*** | 1.20* | 1.22** | 1.60*** | 0.91+ |
|  | (1.39 - 1.64) | (1.17 - 1.41) | (1.15 - 1.30) | (1.18 - 1.62) | (1.03 - 1.39) | (1.08 - 1.37) | (1.45 - 1.77) | (0.82 - 1.01) |
| - Kiel | 1.14** | 1.11* | 1.46*** | 1.32*** | 1.83*** | 1.19** | 1.07 | 0.96 |
|  | (1.05 - 1.24) | (1.01 - 1.23) | (1.37 - 1.56) | (1.12 - 1.54) | (1.55 - 2.16) | (1.05 - 1.34) | (0.97 - 1.18) | (0.87 - 1.07) |
| - Neubrandenburg | 0.96 | 1.26*** | 0.63*** | 1.53*** | 2.36*** | 1.54*** | 1.14** | 0.86** |
|  | (0.89 - 1.03) | (1.15 - 1.37) | (0.59 - 0.66) | (1.31 - 1.77) | (2.01 - 2.78) | (1.38 - 1.73) | (1.04 - 1.25) | (0.78 - 0.95) |
| Number of chronic conditions (count score of 45 chronic conditions) | 1.07*** | 1.10*** | 1.09*** | 1.08*** | 1.06*** | 1.03*** | 1.13*** | 1.11*** |
|  | (1.06 - 1.08) | (1.09 - 1.11) | (1.08 - 1.09) | (1.06 - 1.09) | (1.04 - 1.07) | (1.02 - 1.04) | (1.12 - 1.14) | (1.10 - 1.12) |
| Self-rated health (from 1 = poor to 5 = excellent) | 1.09*** | 1.03* | 1.18*** | 1.16*** | 0.98 | 1.16*** | 1.08*** | 1.14*** |
|  | (1.06 - 1.12) | (1.00 - 1.06) | (1.16 - 1.20) | (1.11 - 1.22) | (0.93 - 1.03) | (1.12 - 1.20) | (1.05 - 1.12) | (1.10 - 1.17) |
|  |  |  |  |  |  |  |  |  |
| Constant | 0.01*** | 0.00*** | 0.02*** | 0.03*** | 0.00*** | 0.15*** | 0.00*** | 0.00*** |
|  | (0.01 - 0.02) | (0.00 - 0.00) | (0.02 - 0.02) | (0.02 - 0.06) | (0.00 - 0.01) | (0.10 - 0.23) | (0.00 - 0.00) | (0.00 - 0.00) |
|  |  |  |  |  |  |  |  |  |
| Observations | 78,449 | 56,324 | 132,298 | 69,804 | 38,571 | 76,124 | 54,268 | 50,448 |
| Pseudo R² | 0.025 | 0.034 | 0.033 | 0.053 | 0.053 | 0.080 | 0.087 | 0.101 |

Notes: Odds Ratios are displayed, 95% CI in parentheses, *** p<0.001, ** p<0.01, * p<0.05, + p<0.10.

Of note, federal voluntary service, voluntary social/ecological year was omitted from the model when X-ray examination of the breast ("mammography", early detection of breast cancer) served as outcome because it predicts the outcome perfectly (7 observations were thus not used).

Supplementary Table 3. Personality and likelihood of cancer screening. Results of adjusted logistic regressions (with standardized personality factors; Germany, between 2014 and 2019)

| Outcomes  Independent variables | Stool examination for blood (haemoccult test, early detection of bowel cancer) - among the total sample | Colonoscopy (screening for colorectal cancer) - among the total sample | Skin examination for moles (early detection of skin cancer) - among the total sample | Breast palpation by a doctor (early detection of breast cancer)  - only among women | X-ray examination of the breast ("mammography", early detection of breast cancer) - only among women | Cervical smear test - only among women | Finger examination of the rectum (early detection of prostate cancer) - only among men | Blood test for prostate cancer (determination of PSA level) - only among men |
| --- | --- | --- | --- | --- | --- | --- | --- | --- |
| Conscientiousness | 1.08*** | 1.04*** | 1.06*** | 1.17*** | 1.15*** | 1.12*** | 1.07*** | 1.10*** |
|  | (1.06 - 1.10) | (1.02 - 1.06) | (1.05 - 1.08) | (1.14 - 1.21) | (1.11 - 1.18) | (1.10 - 1.15) | (1.05 - 1.09) | (1.07 - 1.12) |
| Extraversion | 1.04*** | 1.09*** | 1.04*** | 1.15*** | 1.07*** | 1.08*** | 1.06*** | 1.09*** |
|  | (1.02 - 1.06) | (1.07 - 1.11) | (1.03 - 1.06) | (1.12 - 1.19) | (1.04 - 1.11) | (1.06 - 1.11) | (1.04 - 1.08) | (1.06 - 1.11) |
| Agreeableness | 1.02* | 1.01 | 1.02*** | 1.08*** | 1.07*** | 1.05*** | 1.04*** | 1.04*** |
|  | (1.00 - 1.03) | (0.99 - 1.03) | (1.01 - 1.04) | (1.05 - 1.12) | (1.04 - 1.11) | (1.03 - 1.08) | (1.02 - 1.06) | (1.01 - 1.06) |
| Openness to experience | 1.00 | 0.95*** | 0.97*** | 0.94*** | 0.84*** | 0.97* | 0.97* | 0.96*** |
|  | (0.98 - 1.01) | (0.93 - 0.97) | (0.96 - 0.99) | (0.91 - 0.97) | (0.82 - 0.87) | (0.95 - 0.99) | (0.95 - 1.00) | (0.94 - 0.98) |
| Neuroticism | 1.08*** | 1.12*** | 1.09*** | 1.23*** | 1.12*** | 1.19*** | 1.14*** | 1.14*** |
|  | (1.07 - 1.10) | (1.10 - 1.14) | (1.08 - 1.11) | (1.19 - 1.27) | (1.09 - 1.16) | (1.16 - 1.21) | (1.12 - 1.17) | (1.11 - 1.16) |
| Covariates | ✓ | ✓ | ✓ | ✓ | ✓ | ✓ | ✓ | ✓ |
| Pseudo R² | 0.025 | 0.034 | 0.033 | 0.053 | 0.053 | 0.080 | 0.087 | 0.101 |
| Observations | 78,449 | 56,324 | 132,298 | 69,804 | 38,571 | 76,124 | 54,268 | 50,448 |

Notes: Odds Ratios are displayed, 95% CI in parentheses, *** p<0.001, ** p<0.01, * p<0.05, + p<0.10.

Adjusted for age, sex (if applicable), marital status, education, employment status, study center, income, number of close friends, number of chronic conditions, and self-rated health.

Supplementary Table 4. Effect size measure (Cohen’s d) for personality factors (based on the adjusted logistic regressions presented in Table 3; Germany, between 2014 and 2019)

| Outcomes  Independent variables | Stool examination for blood | Colonoscopy | Skin examination for moles | Breast palpation by a doctor | X-ray examination of the breast | Cervical smear test | Finger examination of the rectum | Blood test for prostate cancer |
| --- | --- | --- | --- | --- | --- | --- | --- | --- |
|  | Effect size (Cohen’s d) | Effect size (Cohen’s d) | Effect size (Cohen’s d) | Effect size (Cohen’s d) | Effect size (Cohen’s d) | Effect size (Cohen’s d) | Effect size (Cohen’s d) | Effect size (Cohen’s d) |
| Conscientiousness | 0.04 | 0.02 | 0.04 | 0.09 | 0.08 | 0.07 | 0.04 | 0.05 |
| Extraversion | 0.02 | 0.04 | 0.02 | 0.06 | 0.03 | 0.04 | 0.03 | 0.04 |
| Agreeableness | 0.01 | 0.01 | 0.01 | 0.05 | 0.04 | 0.03 | 0.02 | 0.02 |
| Openness to experience | 0.00 | -0.02 | -0.01 | -0.03 | -0.07 | -0.01 | -0.01 | -0.02 |
| Neuroticism | 0.03 | 0.04 | 0.04 | 0.08 | 0.05 | 0.07 | 0.05 | 0.05 |

Notes: findings of practical importance (|d|≥.20) would be in bold (if present).

Adjusted for age, sex (if applicable), marital status, education, employment status, study center, income, number of close friends, number of chronic conditions, and self-rated health.

Supplementary Table 5. Personality and likelihood of stool examination for blood. Results of adjusted logistic regressions (personality characteristics were entered separately; Germany, between 2014 and 2019)

| Outcomes  Independent variables | Stool examination for blood (haemoccult test, early detection of bowel cancer) - among the total sample | Stool examination for blood (haemoccult test, early detection of bowel cancer) - among the total sample | Stool examination for blood (haemoccult test, early detection of bowel cancer) - among the total sample | Stool examination for blood (haemoccult test, early detection of bowel cancer) - among the total sample | Stool examination for blood (haemoccult test, early detection of bowel cancer) - among the total sample |
| --- | --- | --- | --- | --- | --- |
| Conscientiousness | 1.08*** |  |  |  |  |
|  | (1.07 - 1.10) |  |  |  |  |
| Extraversion |  | 1.03*** |  |  |  |
|  |  | (1.02 - 1.04) |  |  |  |
| Agreeableness |  |  | 1.02** |  |  |
|  |  |  | (1.01 - 1.04) |  |  |
| Openness to experience |  |  |  | 1.02** |  |
|  |  |  |  | (1.01 - 1.03) |  |
| Neuroticism |  |  |  |  | 1.05*** |
|  |  |  |  |  | (1.03 - 1.06) |
| Covariates | ✓ | ✓ | ✓ | ✓ | ✓ |
| Pseudo R² | 0.024 | 0.023 | 0.023 | 0.023 | 0.023 |
| Observations | 78,450 | 78,449 | 78,449 | 78,449 | 78,449 |

Notes: Odds Ratios are displayed, 95% CI in parentheses, *** p<0.001, ** p<0.01, * p<0.05, + p<0.10.

Adjusted for age, sex, marital status, education, employment status, study center, income, number of close friends, number of chronic conditions, and self-rated health.

Supplementary Table 6. Personality and likelihood of colonoscopy. Results of adjusted logistic regressions (personality characteristics were entered separately; Germany, between 2014 and 2019)

| Outcomes  Independent variables | Colonoscopy (screening for colorectal cancer) - among the total sample | Colonoscopy (screening for colorectal cancer) - among the total sample | Colonoscopy (screening for colorectal cancer) - among the total sample | Colonoscopy (screening for colorectal cancer) - among the total sample | Colonoscopy (screening for colorectal cancer) - among the total sample |
| --- | --- | --- | --- | --- | --- |
| Conscientiousness | 1.04*** |  |  |  |  |
|  | (1.02 - 1.06) |  |  |  |  |
| Extraversion |  | 1.04*** |  |  |  |
|  |  | (1.03 - 1.06) |  |  |  |
| Agreeableness |  |  | 1.00 |  |  |
|  |  |  | (0.98 - 1.02) |  |  |
| Openness to experience |  |  |  | 0.99+ |  |
|  |  |  |  | (0.97 - 1.00) |  |
| Neuroticism |  |  |  |  | 1.07*** |
|  |  |  |  |  | (1.06 - 1.08) |
| Covariates | ✓ | ✓ | ✓ | ✓ | ✓ |
| Pseudo R² | 0.032 | 0.032 | 0.032 | 0.032 | 0.033 |
| Observations | 56,325 | 56,324 | 56,324 | 56,324 | 56,324 |

Notes: Odds Ratios are displayed, 95% CI in parentheses, *** p<0.001, ** p<0.01, * p<0.05, + p<0.10.

Adjusted for age, sex, marital status, education, employment status, study center, income, number of close friends, number of chronic conditions, and self-rated health.

Supplementary Table 7. Personality and likelihood of skin examination for moles. Results of adjusted logistic regressions (personality characteristics were entered separately; Germany, between 2014 and 2019)

| Outcomes  Independent variables | Skin examination for moles (early detection of skin cancer) - among the total sample | Skin examination for moles (early detection of skin cancer) - among the total sample | Skin examination for moles (early detection of skin cancer) - among the total sample | Skin examination for moles (early detection of skin cancer) - among the total sample | Skin examination for moles (early detection of skin cancer) - among the total sample |
| --- | --- | --- | --- | --- | --- |
| Conscientiousness | 1.06*** |  |  |  |  |
|  | (1.05 - 1.08) |  |  |  |  |
| Extraversion |  | 1.02*** |  |  |  |
|  |  | (1.01 - 1.03) |  |  |  |
| Agreeableness |  |  | 1.02** |  |  |
|  |  |  | (1.01 - 1.03) |  |  |
| Openness to experience |  |  |  | 1.00 |  |
|  |  |  |  | (0.99 - 1.01) |  |
| Neuroticism |  |  |  |  | 1.05*** |
|  |  |  |  |  | (1.04 - 1.06) |
| Covariates | ✓ | ✓ | ✓ | ✓ | ✓ |
| Pseudo R² | 0.032 | 0.032 | 0.032 | 0.032 | 0.032 |
| Observations | 132,299 | 132,298 | 132,298 | 132,298 | 132,298 |

Notes: Odds Ratios are displayed, 95% CI in parentheses, *** p<0.001, ** p<0.01, * p<0.05, + p<0.10.

Adjusted for age, sex, marital status, education, employment status, study center, income, number of close friends, number of chronic conditions, and self-rated health.

Supplementary Table 8. Personality and likelihood of breast palpation by a doctor. Results of adjusted logistic regressions (personality characteristics were entered separately; Germany, between 2014 and 2019)

| Outcomes  Independent variables | Breast palpation by a doctor (early detection of breast cancer)  - only among women | Breast palpation by a doctor (early detection of breast cancer)  - only among women | Breast palpation by a doctor (early detection of breast cancer)  - only among women | Breast palpation by a doctor (early detection of breast cancer)  - only among women | Breast palpation by a doctor (early detection of breast cancer)  - only among women |
| --- | --- | --- | --- | --- | --- |
| Conscientiousness | 1.19*** |  |  |  |  |
|  | (1.16 - 1.23) |  |  |  |  |
| Extraversion |  | 1.09*** |  |  |  |
|  |  | (1.07 - 1.12) |  |  |  |
| Agreeableness |  |  | 1.09*** |  |  |
|  |  |  | (1.06 - 1.12) |  |  |
| Openness to experience |  |  |  | 1.01 |  |
|  |  |  |  | (0.99 - 1.03) |  |
| Neuroticism |  |  |  |  | 1.11*** |
|  |  |  |  |  | (1.09 - 1.14) |
| Covariates | ✓ | ✓ | ✓ | ✓ | ✓ |
| Pseudo R² | 0.048 | 0.045 | 0.045 | 0.044 | 0.046 |
| Observations | 69,805 | 69,804 | 69,804 | 69,804 | 69,804 |

Notes: Odds Ratios are displayed, 95% CI in parentheses, *** p<0.001, ** p<0.01, * p<0.05, + p<0.10.

Adjusted for age, marital status, education, employment status, study center, income, number of close friends, number of chronic conditions, and self-rated health.

Supplementary Table 9. Personality and likelihood of X-ray examination of the breast. Results of adjusted logistic regressions (personality characteristics were entered separately; Germany, between 2014 and 2019)

| Outcomes  Independent variables | X-ray examination of the breast ("mammography", early detection of breast cancer) - only among women | X-ray examination of the breast ("mammography", early detection of breast cancer) - only among women | X-ray examination of the breast ("mammography", early detection of breast cancer) - only among women | X-ray examination of the breast ("mammography", early detection of breast cancer) - only among women | X-ray examination of the breast ("mammography", early detection of breast cancer) - only among women |
| --- | --- | --- | --- | --- | --- |
| Conscientiousness | 1.13*** |  |  |  |  |
|  | (1.10 - 1.17) |  |  |  |  |
| Extraversion |  | 1.01 |  |  |  |
|  |  | (0.98 - 1.03) |  |  |  |
| Agreeableness |  |  | 1.07*** |  |  |
|  |  |  | (1.03 - 1.10) |  |  |
| Openness to experience |  |  |  | 0.91*** |  |
|  |  |  |  | (0.89 - 0.93) |  |
| Neuroticism |  |  |  |  | 1.06*** |
|  |  |  |  |  | (1.04 - 1.09) |
| Covariates | ✓ | ✓ | ✓ | ✓ | ✓ |
| Pseudo R² | 0.048 | 0.046 | 0.046 | 0.048 | 0.047 |
| Observations | 38,572 | 38,571 | 38,571 | 38,571 | 38,571 |

Notes: Odds Ratios are displayed, 95% CI in parentheses, *** p<0.001, ** p<0.01, * p<0.05, + p<0.10.

Adjusted for age, marital status, education, employment status, study center, income, number of close friends, number of chronic conditions, and self-rated health.

Supplementary Table 10. Personality and likelihood of Cervical smear test. Results of adjusted logistic regressions (personality characteristics were entered separately; Germany, between 2014 and 2019)

| Outcomes  Independent variables | Cervical smear test - only among women | Cervical smear test - only among women | Cervical smear test - only among women | Cervical smear test - only among women | Cervical smear test - only among women |
| --- | --- | --- | --- | --- | --- |
| Conscientiousness | 1.13*** |  |  |  |  |
|  | (1.11 - 1.16) |  |  |  |  |
| Extraversion |  | 1.05*** |  |  |  |
|  |  | (1.03 - 1.07) |  |  |  |
| Agreeableness |  |  | 1.05*** |  |  |
|  |  |  | (1.03 - 1.08) |  |  |
| Openness to experience |  |  |  | 1.01 |  |
|  |  |  |  | (0.99 - 1.03) |  |
| Neuroticism |  |  |  |  | 1.10*** |
|  |  |  |  |  | (1.08 - 1.12) |
| Covariates | ✓ | ✓ | ✓ | ✓ | ✓ |
| Pseudo R² | 0.076 | 0.074 | 0.074 | 0.074 | 0.076 |
| Observations | 76,125 | 76,124 | 76,124 | 76,124 | 76,124 |

Notes: Odds Ratios are displayed, 95% CI in parentheses, *** p<0.001, ** p<0.01, * p<0.05, + p<0.10.

Adjusted for age, marital status, education, employment status, study center, income, number of close friends, number of chronic conditions, and self-rated health.

Supplementary Table 11. Personality and likelihood of Finger examination of the rectum. Results of adjusted logistic regressions (personality characteristics were entered separately; Germany, between 2014 and 2019)

| Outcomes  Independent variables | Finger examination of the rectum (early detection of prostate cancer) | Finger examination of the rectum (early detection of prostate cancer) | Finger examination of the rectum (early detection of prostate cancer) | Finger examination of the rectum (early detection of prostate cancer) | Finger examination of the rectum (early detection of prostate cancer) |
| --- | --- | --- | --- | --- | --- |
| Conscientiousness | 1.07*** |  |  |  |  |
|  | (1.05 - 1.09) |  |  |  |  |
| Extraversion |  | 1.04*** |  |  |  |
|  |  | (1.02 - 1.05) |  |  |  |
| Agreeableness |  |  | 1.03** |  |  |
|  |  |  | (1.01 - 1.05) |  |  |
| Openness to experience |  |  |  | 1.01 |  |
|  |  |  |  | (0.99 - 1.02) |  |
| Neuroticism |  |  |  |  | 1.08*** |
|  |  |  |  |  | (1.06 - 1.09) |
| Covariates | ✓ | ✓ | ✓ | ✓ | ✓ |
| Pseudo R² | 0.085 | 0.084 | 0.084 | 0.084 | 0.085 |
| Observations | 54,268 | 54,268 | 54,268 | 54,268 | 54,268 |

Notes: Odds Ratios are displayed, 95% CI in parentheses, *** p<0.001, ** p<0.01, * p<0.05, + p<0.10.

Adjusted for age, marital status, education, employment status, study center, income, number of close friends, number of chronic conditions, and self-rated health.

Supplementary Table 12. Personality and likelihood of Blood test for prostate cancer. Results of adjusted logistic regressions (personality characteristics were entered separately; Germany, between 2014 and 2019)

| Outcomes  Independent variables | Blood test for prostate cancer (determination of PSA level) - only among men | Blood test for prostate cancer (determination of PSA level) - only among men | Blood test for prostate cancer (determination of PSA level) - only among men | Blood test for prostate cancer (determination of PSA level) - only among men | Blood test for prostate cancer (determination of PSA level) - only among men |
| --- | --- | --- | --- | --- | --- |
| Conscientiousness | 1.10*** |  |  |  |  |
|  | (1.08 - 1.12) |  |  |  |  |
| Extraversion |  | 1.06*** |  |  |  |
|  |  | (1.04 - 1.07) |  |  |  |
| Agreeableness |  |  | 1.03** |  |  |
|  |  |  | (1.01 - 1.05) |  |  |
| Openness to experience |  |  |  | 1.01 |  |
|  |  |  |  | (0.99 - 1.02) |  |
| Neuroticism |  |  |  |  | 1.07*** |
|  |  |  |  |  | (1.05 - 1.09) |
| Covariates | ✓ | ✓ | ✓ | ✓ | ✓ |
| Pseudo R² | 0.099 | 0.098 | 0.097 | 0.097 | 0.098 |
| Observations | 50,448 | 50,448 | 50,448 | 50,448 | 50,448 |

Notes: Odds Ratios are displayed, 95% CI in parentheses, *** p<0.001, ** p<0.01, * p<0.05, + p<0.10.

Adjusted for age, marital status, education, employment status, study center, income, number of close friends, number of chronic conditions, and self-rated health.
